# Multielement stoichiometry of submerged macrophytes across Yunnan plateau lakes (China)

**DOI:** 10.1038/srep10186

**Published:** 2015-05-13

**Authors:** Wei Xing, Haoping Wu, Qiao Shi, Beibei Hao, Han Liu, Zhixiu Wang, Guihua Liu

**Affiliations:** 1 Key Laboratory of Aquatic Botany and Watershed Ecology, Wuhan Botanical Garden, Chinese Academy of Sciences, Wuhan 430074, China; 2 University of Chinese Academy of Sciences, Beijing 100049, China

## Abstract

Stoichiometric homeostasis of element composition is one of the central concepts of ecological stoichiometry. We analyzed concentrations of macroelements (C, N, P, Ca, K, Mg, S), microelements (Cu, Fe, Mn, Mo, Ni, Zn) and beneficial elements (Na, Se, Si) in submerged macrophytes, water and sediments across 20 Yunnan plateau lakes. We predicted that tissue element composition in submerged macrophytes is affected by lake trophic level and taxonomy, and submerged macrophytes have weak stoichiometric homeostasis for all above 16 elements. Canonical discriminant analyses successfully discriminated among trophic level groups and taxa groups. Of all the elements, C, N, P and S most effectively discriminated among trophic level groups across 20 lakes, revealing lake trophic level mostly affect tissue macroelement composition in submerged macrophytes; while Ca, K and Se most effectively discriminated among submerged macrophytes taxa groups, suggesting taxonomy mostly affect compositions of macroelements and beneficial elements in submerged macrophytes. In addition, the stoichiometric homeostatic coefficient of 1/*H*_Ca:C_ for all five taxa of submerged macrophytes were less than zero, suggesting submerged macrophytes in Yunnan plateau lakes have strong Ca stoichiometric homeostasis. Our findings, not only broaden the knowledge of multielement stoichiometric homeostasis, but also help to choose most appropriate lake management strategy.

Ecological stoichiometry is a powerful approach for examining the balance of multielement in ecological processes^1^. This approach has pushed us for better understanding of trophic transfer and nutrient cycling in aquatic and terrestrial ecosystems^2^,^3^,^4^,^5^. It is well established that all plant species require at least 16 elements for their growth and survival, but the primary focus lies on three macroelements— C, N and P in studies on ecological stoichiometry^6^. Consequently, the application of ecological stoichiometry to other macroelements (Ca, K, Mg and S), essential microelements (Cu, Fe, Mn, Mo, Ni and Zn) and beneficial elements (Na, Se and Si) is relatively scarce^7^,^8^,^9^.

Stoichiometric homeostasis of element composition is one of the central concepts of ecological stoichiometry^1^,^10^. Autotrophic organisms are normally considered to be non-homeostatic because their element composition can vary widely with fluctuations of their surrounding environments, whereas heterotrophs are generally thought to be strictly homeostatic^1^,^10^. Plant stoichiometry varies with growth rate and the surrounding environment^6^. Therefore, the balance of multielement in plants plays a pivotal role in plant growth^7^,^11^. A large amount of studies have documented that stoichiometric homeostasis of plants is lower than animals and bacteria, but higher than plankton and fungi^1^,^12^,^13^,^14^,^15^. Though C, N and P stoichiometric homeostasis of organisms in aquatic and terrestrial ecosystems have been studied extensively^4^,^5^,^8^,^10^,^16^,^17^,^18^, little is known about stoichiometric homeostasis of other elements mentioned above in organisms^7^,^8^,^19^. The ecological stoichiometry provides an effective approach to understand patterns of cycling, limitation and excess accumulation of these less-studied elements in natural organism populations^8^.

It is well documented that environmental stoichiometry can strongly affect stoichiometry of organisms^3^,^20^,^21^,^22^, which may cause imbalance of elements in organisms^11^,^23^. Consequently, resulting changes in physiological and ecological processes can further lead to shifts of species composition or loss of some species^5^,^24^. Sardans *et al.*^25^ pointed out that N deposition increases the N:P ratio in the plants of terrestrial and freshwater ecosystems, reducing soil and water N_2_ fixation capacity and ecosystem species diversity. Zhang *et al.*^19^ further reported that changes in environmental factors (precipitation and temperature) can affect plant leaf element (N, P, K, S, Ca, Fe, Mn, Si, Na and Al) composition and terrestrial ecosystem functioning.

Many studies have shown that significant variations in plant element concentration can be explained by taxonomic affiliation^19^,^26^,^27^. Plant taxa may differ in the need for, and capability of obtaining and maintaining, specific ranges of concentrations and ratios of different elements in the plant body^1^,^7^. Previous studies showed that plants in different angiosperm families and orders may accumulate significantly different concentrations of Ca, K and Mg in their shoots; eudicots generally have higher shoot Ca concentration than monocots, while commelinoid monocot species have lower shoot Ca concentration than other monocot species^26^,^28^,^29^. However, little is known about effects of taxonomy on multielement composition in submerged macrophytes.

Submerged macrophytes have important structural and functional effects on aquatic ecosystems^30^, which can improve water quality through various mechanisms^31^,^32^. However, element concentrations and stoichiometry of submerged macrophytes in lakes has received less attention^3^, especially in plateau lakes. We hypothesized that tissue element stoichiometry of submerged macrophytes is affected by lake trophic level (represented by N and P concentrations in water and sediments) and taxonomy, and submerged macrophytes have weak stoichiometric homeostasis (strong plastic) for all above 16 elements. The hypothesis contains three questions: 1) Do environmental N and P concentrations affect elemental composition of submerged macrophytes? 2) Does elemental composition differ among submerged macrophytes taxa? 3) Are submerged macrophytes non-homeostatic for all 16 elements? Therefore, in the study, we want to answer questions and further test the hypothesis by determining the 16 elements concentrations in submerged macrophytes and their surrounding environments (water and sediments) across 20 Yunnan plateau lakes.

## Results

### Collected species and tissue multielement ratios in submerged macrophytes

The species composition of submerged macrophytes was significantly different among most of lakes (Table S1). We totally collected 16 species of submerged macrophytes in 20 Yunnan plateau lakes. They belong to five taxa: Potamogetonaceae (9 species, *Potamogeton malaianus* Miq., *Potamogeton pectinatus* L., *Potamogeton maackianus* A. Benn., *Potamogeton perfoliatus* L., *Potamogeton lucens* L., *Potamogeton crispus* L., *Potamogeton pusillus* L., *Potamogeton distinctus* A. Benn. and *Potamogeton compressus* Linn.), Hydrocharitaceae (4 species, *Hydrilla verticillata* (L.f.) Royle, *Najas marina* L., *Ottelia acuminata* (Gagnep.) Dandy and *Vallisneria natans* (Lour.) H. Hara), Haloragaceae (*Myriophyllum spicatum* L.), Ceratophyllaceae (*Ceratophyllum demersum* L.) and Characeae (*Chara sp.*).

**Figure 1:**
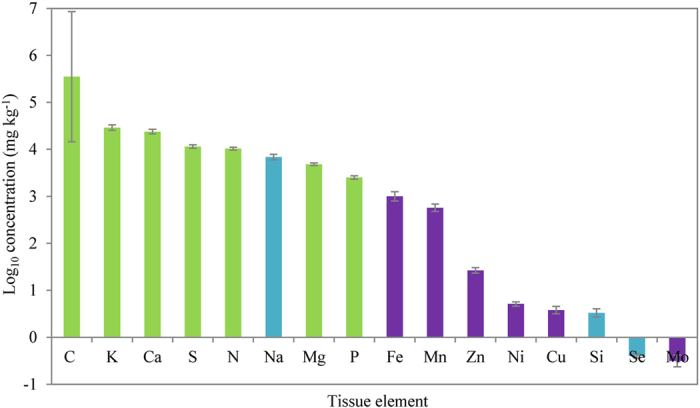
The average concentration of 16 tissue elements in submerged macrophytes across 20 Yunnan plateau lakes. These data were log_10_-transformed and represented as means ± SE. Macroelements were colored green, beneficial elements were colored blue, and microelements were colored purple.

The average concentrations of 16 tissue elements in submerged macrophytes across 20 Yunnan plateau lakes varied widely (Fig. 1). The order of the average element concentration was C>K>Ca>S>N>Na>Mg>P>Fe>Mn>Zn>Ni>Cu>Si>Se>Mo. The average mass ratios of C:K:Ca:S:N:Na:Mg:P:Fe:Mn and Zn:Ni:Cu:Si:Se:Mo were 133:13:10:5:4:3:2:1:0.6:0.3 and 76:14:14:14:1:1, respectively.

### Effect of lake trophic level on multielement composition in submerged macrophytes

**Figure 2:**
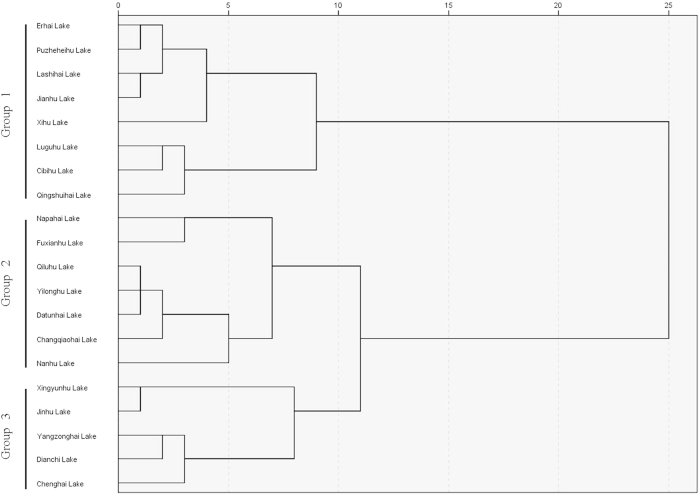
Hierarchical clustering analysis of 20 Yunnan plateau lakes based on environmental nutrients (water total nitrogen, water total phosphorus, water total dissolved nitrogen, water total dissolved phosphorus (mg L^−1^) and sediment total nitrogen, sediment nitrate, sediment ammonia, sediment total phosphorus and sediment dissolved phosphorus (mg kg^−1^)). This distance is based on the Pearson correlation coefficient.

We classified 20 lakes into three trophic level groups by clustering analysis based on environmental nutrients concentrations (water total nitrogen, water total phosphorus, water total dissolved nitrogen, water total dissolved phosphorus (mg L^−1^) and sediment total nitrogen, sediment nitrate, sediment ammonia, sediment total phosphorus and sediment dissolved phosphorus (mg kg^−1^)). There are 8 lakes in group 1 including Erhai Lake, Puzhehei Lake, Lashihai Lake, Jianhu Lake, Xihu Lake, Luguhu Lake, Cibihu Lake and Qingshuihai Lake, 7 lakes in group 2 including Napahai Lake, Fuxianhu Lake, Qiluhu Lake, Yilonghu Lake, Datunhai Lake, Changqiaohai Lake and Nanhu Lake, and 5 lakes in groups 3 including Xingyunhu Lake, Jinhu Lake, Yangzonghai Lake, Dianchi Lake and Chenghai lake (Fig. 2).

**Figure 3:**
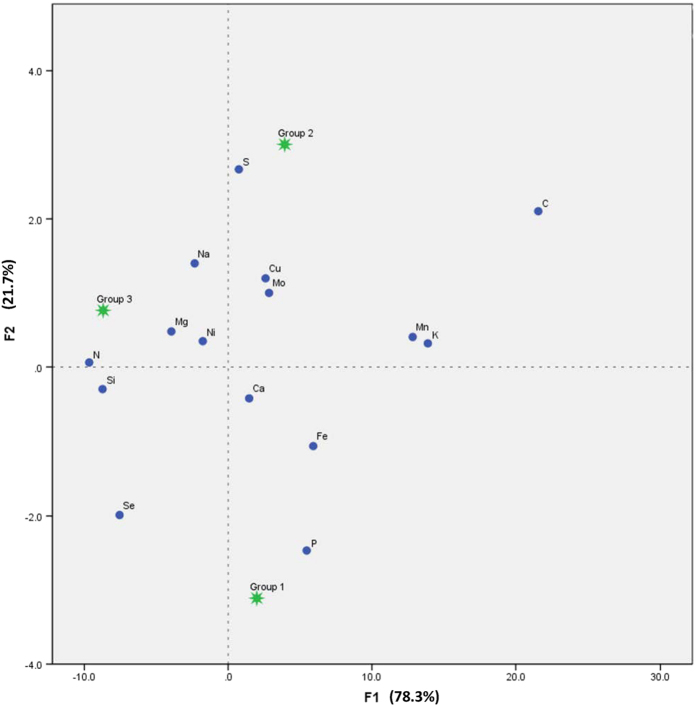
Difference of tissue multielement composition among lake trophic level groups by canonical discriminant analysis (F1: standard canonical function, F2: group centroid function).

Canonical discriminant analysis successfully discriminated among three lake trophic level groups (Fig. 3). In lake trophic level groups, the first two canonical discriminant functions, F1 and F2, explained complete variations (F1, 78.3%; F2, 21.7%) in element composition. Unfortunately, tissue Zn was not included in the CDA of lake trophic level groups because of tolerance limit (<0.001). Figure 3 showed that P, S and N were the most effective element in CDA among lakes. Phosphorus, Fe and Ca discriminated lake group 1, S, Cu, Mo and C discriminated lake group 2, and N, Mg and Ni discriminated lake group 3.

### Differences in multielement composition among submerged macrophytes taxa

**Figure 4:**
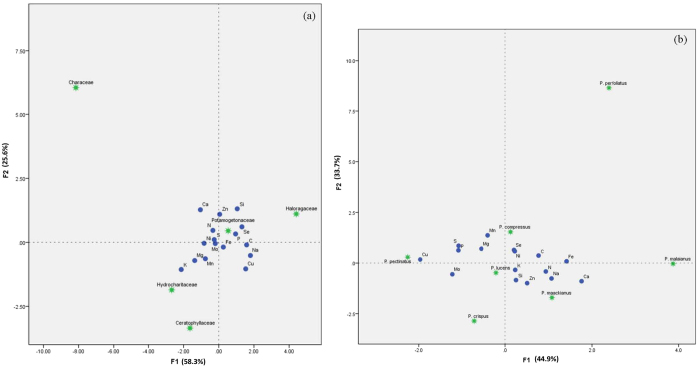
Differences of tissue multielement composition among submerged macrophytes taxa and Potamogetonaceae taxa by canonical discriminant analysis (F1: standard canonical function, F2: group centroid function). (**a**) Submerged macrophytes taxa, (**b**) Potamogetonaceae taxa.

We classified submerged macrophytes into five taxa groups: Potamogetonaceae, Hydrocharitaceae, Haloragaceae, Ceratophyllaceae and Characeae. Canonical discriminant analysis also successfully discriminated among five taxa groups (Fig. 4a). In submerged macrophytes taxa groups, the first two canonical discriminant functions, F1 and F2, explained the majority of variation (F1, 58.3%; F2, 25.6%) in element composition. Fig. 4a showed that Ca, K, and Si were the most effective element in CDA among submerged macrophytes taxa. Calcium and N discriminated Characeae, Si, Se and P discriminated Halogaceae, and K, Mg and Mn discriminated Hydrochritaceae and Ceratophyllaceae. Notably, Potamogetonaceae in the CDA plot was very close to central point (Fig. 4a). Therefore, we performed a further CDA to explore the differences in multielement composition within Patamogetonaceae taxa (Fig. 4b). However, *P. pusillus* and *P. distinctus* were not used in above CDA because of low frequency (less than 3 times) in all sampled sites. In Patamogetonaceae taxa, the first two canonical discriminant functions, F1 and F2, explained the majority of variation (F1, 44.9%; F2, 33.7%) in element composition. Result of fig. 4 indicated that Cu, Se, Fe, Ca, Na and Mo were the most effective element in CDA among Patamogetonaceae taxa. Copper, P, Mo and S discriminated species *P. pectinatus*, Se and Ni discriminated species P*. compress* and *P. perfoliatus*, Fe, Ca and Na discriminated species *P. malaianus*, Na and Zn discriminated species *P. maackianus*, Mo and Si discriminated species *P. cripus*, while species *P. lucens* didn’t successfully discriminated by these 16 elements.

### Elemental stoichiometric homeostasis in submerged macrophytes taxa

Table 1 showed that elemental stoichiometric homeostasis of submerged macrophytes appears polarization. The stoichiometric homeostasis coefficients (1/*H*_Ca:C_) for all five taxa of submerged macrophytes were less than zero, and the order was Potamogetonaceae (− 4.52) > Hydrocharitaceae (−3.47) > Haloragaceae (−2.81) > Ceratophyllaceae (−1.97) > Characeae (−1.79) (Table 1). For other elements beside Na and Fe, homeostatic coefficients (1/*H*_X:C_) for submerged macrophytes were all more than 0.75. Homeostatic coefficients of 1/*H*_Na:C_ for Potamogetonaceae and Hydrocharitaceae were −0.14 and −2.08, respectively.

## Discussion

### Multielement stoichiometric ratios in tissues of submerged macrophytes

It is necessary to maintain sufficient concentrations and relatively stable nutrient ratios in plant tissues (stoichiometric balance) for healthy growth^7^. In the study, the average mass ratios of C:K:Ca:S:N:Na:Mg:P:Fe:Mn and Zn:Ni:Cu:Si:Se:Mo in submerged macrophytes were 133:13:10:5:4:3:2:1:0.6:0.3 and 76:14:14:14:1:1, respectively. To our knowledge, there are very few studies concerning multielement stoichiometry of plants. Han *et al.*^7^ reported that elemental ratios of N:P:K:Ca:Mg:S:Si:Fe:Na:Mn:Al in leaves of China’s terrestrial plants is 100:6.8:50:43:11:7.7:19:1.4:7:0.54:2.2. Wang and Moore^33^ pointed out that the average mass ratios of C:N:P:K in the foliar tissues from a Canadian peatland is 445:14:1:9. The differences of element ratios in submerged macrophytes, terrestrial plants and peatland plants suggested that species composition and environmental element ratios interact tightly^1^,^20^. The interaction led to certain element composition (ratio) in plants.

### Lake trophic level affects element composition of submerged macrophytes

Increasing inputs of N and P nutrients can cause lake eutrophication which brings about changes in water chemistry, including pH, dissolved O_2_ (DO), oxidation-reduction potential (ORP) and other chemical factors^34^,^35^. Consequently, these changes alter bioavailability and chemical forms of chemical elements, resulting in variations of nutrients uptake by organisms^36^,^37^. In this study, canonical discriminant analysis successfully discriminated among three trophic level groups, revealing that multielement concentrations and stoichiometry of submerged macrophytes are markedly affected by lake trophic level (Fig. 3). Of all the elements, macroelements (C, N, P, S and Mg) most effectively discriminated among lake trophic level groups across 20 plateau lakes, suggesting lake trophic level mostly affect tissue macroelement composition in submerged macrophytes.

In fig. 3, we found that close relationships of Lake group 1 and tissue P, and Lake group 3 and tissue N, suggesting tissue P and tissue N in submerged macrophytes can be easily affected by high environmental N and P. The reason may be related to tissue critical nutrient thresholds^38^ and homeostasis^1^. Gerloff 38 reported that the N and P critical nutrient concentration for 95% maximum growth P = 0.16 (±0.15 SD) % DW (dry weight) and N = 1.82 (±0.62 SD)% DW. However, in this study the average tissue concentrations of N and P were 1.08% DW and 0.26% DW, respectively, and N:P ratio was only about 4, suggesting submerged macrophytes are N-limited and P-rich in Yunnan plateau lakes according to Koerselman and Meuleman^39^. It is well documented that biomass N:P ratios can be used to examine how the N and P influence the plant species composition or various ecological processes^20^. In Yunnan plateau lakes, increase of P caused relatively deficiency of N in submerged macrophytes according to stoichiometric balance^1^. Most importantly, the P pollution in Yunnan Plateau lakes is very serious because of excess exploitation of P and other metal mines. Our result was contrary to Elser *et al.*^40^ and Sardans *et al.*^25^ that high N supply in lakes leads to P-limited in organisms, which is caused by both limited environmental N and P in their studied lakes. In addition, high N trophic level can also influence tissue Fe and Ca, and high P trophic level can influence tissue N, Mg, Na and Ni. To our knowledge, it is the first report to study whether trophic level affects multielement composition (stoichiometry) of submerged macrophytes in plateau lakes.

### Taxonomy affects element composition of submerged macrophytes

Considerable previous studies have confirmed that leaf heavy metal concentrations, shoot mineral concentrations, and N, P and Mg concentrations in plant organs are constrained by taxonomic affiliations^19^,^26^,^41^. In this study, canonical discriminant analysis also successfully discriminated among five taxa groups, revealing that submerged macrophytes have unique multielement composition (Fig. 4a). Of all the elements, Ca, K and Se most effectively discriminated among submerged macrophytes taxa groups, which is not completely in line with the study of Karimi and Folt^8^. The reason may be caused by difference of elemental stoichiometry between submerged macrophytes taxa and invertebrates taxa. Demars and Edwards^42^ and Li *et al.*^43^ also pointed out that species identity (taxon) could explain the majority of the variances in aquatic plant tissue nutrient (N and P) concentrations.

Our result showed that Cu, Se, Fe, Ca, Na and Mo were the most effective element in CDA among Patamogetonaceae taxa (Fig. 4b), suggesting trace elements play pivotal role in charactering species identity within a specific taxon. The CDA biplot of Patamogetonaceae taxa can visually distinguish the differences of multielement composition among species, such as *P. pectinatus* and *P. malaianus*, and *P. perfoliatus* and *P. crispus*. Our result also indicated that *P. lucens* is the best species for ecological restoration of Yunnan plateau lakes because of high stoichiometric homeostasis (no change in elemental composition).

### Strong Ca stoichiometric homeostasis in submerged macrophytes

Homeostasis represents the ability of an organism to maintain a given element ratio in the body despite fluctuations of the surrounding environment^1^. Knowledge of the degree of stoichiometric homeostasis in plants is important because it underpins our interpretation of observed variation in plant elemental stoichiometric ratios in nature^1^. Carbon, N and P stoichiometric homeostasis of organisms is the most widely studied topic in a variety of ecosystems^4^,^10^,^17^. Demars and Edwards^42^and Li *et al.*^43^ both reported strict C:N:P stoichiometric ratios for freshwater aquatic macrophytes. But we didn’t find strong stoichiometric homeostasis for C, N and P by classical equation provided by Sterner and Elser^1^ in the study. The homeostasis parameter, 1/*H* is a useful tool that used in the study to quantify the stoichiometric homeostasis of submerged macrophytes^10^. The result of strict C:N:P homeostasis obtained by Demars and Edwards^42^ and Li *et al.*^43^ was not from the calculation of classic equation, but from inference of element ratios. Compared with previous studies, stoichiometric homeostasis coefficients for submerged macrophytes in the study were less than animals, invertebrates and zooplankton, even fungi and algae^10^. But it is well documented that stoichiometric homeostasis of plants is stronger than that of algae and fungi and weaker than that of animals^1^.

Beside C, N and P, other elements homeostasis of organisms has received less attention^8^. Karimi and Folt^8^ pointed out that homeostatic coefficients for freshwater invertebrates were highest for macronutrients, intermediated for essential micronutrients and low for non-essential metals, which is not consistent with our results. Our results showed that Ca and Na stoichiometric homeostasis coefficients for submerged macrophytes are relatively higher than other elements, especially C, N and P. In the study, elemental stoichiometric homeostasis of submerged macrophytes appeared polarization (Table 1). Calcium homeostasis coefficients (1/*H*_Ca:C_) for all submerged macrophytes taxa and Na homeostasis coefficients (1/*H*_Na:C_) for Potamogetonaceae taxa and Hydrocharitaceae taxa are less than zero, while other elements homeostasis coefficients are more than 0.75. The reason is most probably luxury uptake and storage of nutrients by submerged macrophytes (species identity)^44^,^45^. In general, stoichiometric homeostasis of submerged macrophytes depends on external (environment) and internal (species identity) factors. This is also the first comprehensive evaluation of factors influencing stoichiometric homeostasis in submerged macrophytes for multielement.

To our knowledge, our study is the first to comprehensively document the tissue multielement (16 elements) compositions and stoichiometric homeostasis of submerged macrophytes in plateau lakes. We successfully tested that multielement stoichiometry in tissues of submerged macrophytes is affected by lake trophic level and taxonomy. As Karimi and Folt^8^ said, CDAs are particularly effective at revealing taxonomic differences in stoichiometry of multielement. In this study, CDAs indeed successfully discriminated among lake trophic level and submerged macrophytes taxa based on multielement composition in lake trophic level groups and taxonomic groups. Our findings, not only broaden the knowledge of multielement stoichiometric homeostasis, but also help us to choose the most appropriate lake management strategy, e.g., to maintain and restore certain types of macrophytes. We also successfully tested that submerged macrophytes have weak elemental stoichiometric homeostasis (strong plastic), but not for all 16 elements. Submerged macrophytes in Yunnan plateau lakes have strong Ca stoichiometric homeostasis. From the study, we think confidently that the homeostasis parameter 1/*H* is very adaptable for submerged macrophytes, and restoration of aquatic plants must followed local principles, e.g., choosing native aquatic macrophytes. In conclusion, stoichiometric homeostasis of submerged macrophytes depends on external (environment) and internal (species identity) factors.

## Methods

### Study area

The Yunnan Plateau is located in southwest China and covers an area of 394,000 km^2^. This plateau is the middle tier on the eastern slope of the Himalayas and has intensive neotectonic movement since late Pliocene time^46^. Therefore, there are large amounts of lakes on this plateau. In this study, we selected 20 lakes with different trophic level across the plateau. Locations and limnological characteristics of 20 lakes are presented in Fig. S1 and table S2, respectively.

### Field sampling

We conducted the study in May, 2012. Samples were collected within 1 month to minimize confounding temporal with spatial variation^8^. We collected above-ground parts of submerged macrophytes and put them into respective cloth bags with waterproof labels after species identification. We also collected corresponding water and sediments for element determination with polyethylene bottles and valve bags, respectively.

### Laboratory analysis

We thoroughly rinsed and cleaned the collected submerged macrophytes to completely remove sediments, algae and invertebrates. Samples of submerged macrophytes and sediments were dried for 48 h at 80 ^o^C and for 24 h at 105 ^o^C, respectively, and were ground and homogenized before digestion. An accurately weighed plant or sediment sample was placed in a Teflon nitrification tank, 6.0 mL HNO_3_, 0.5 mL HCl, and 3.0 mL HF were added^47^. The sealed tank was then placed in a microwave oven and nitrified at 180 ± 5 °C for 15 min^48^. The residue from the tank was then transferred into a Teflon breaker and dissolved with 0.5 mL HClO_4_ in a heating block at about 200 °C and diluted to 25 mL with double distilled deionized water. Water samples were filtered through a 0.45 μm cellulose acetate membrane before measurement.

Total carbon (TC) concentrations were measured by TOC analyzer (Multi N/C 2100, Jena, Germany). Total nitrogen (TN) concentrations were analyzed using the micro-Kjeldahl method^49^. Total phosphorus (TP) concentrations were measured by the ammonium molybdate method after persulfate oxidation^50^. Other 13 elements (Ca, Cu, Fe, K, Mg, Mn, Mo, Na, Ni, S, Se, Si, Zn) in above solutions were detected using inductively coupled plasma-atomic emission spectrometry (ICP-AES) (IRIS Intrepid II XSP, Thermo Elemental, USA)^45^.

### Calculating stoichiometric homeostasis coefficients

Stoichiometric homeostasis coefficients (1/*H*) were calculating according to the following equation,
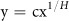
where y is the tissue element stoichiometry of submerged macrophytes (X:C), x is the element stoichiometry of corresponding environment (X:C in water + sediment), c is a constant and 1/*H* is the slope of the log-linearized relationship^1^. In this study, the slope, 1/*H*, was used because strictly homeostatic organisms have an *H* of infinity presenting a number of analytic problems^10^. According to Makino^12^ and Persson^10^, the degree of homeostasis of species was classified as: 1/*H* ≤ 0, strict homeostatic; 0 < 1/*H* < 0.25, homeostatic; 0.25 < 1/*H* < 0.5, weakly homeostatic; 0.5 < 1/*H* < 0.75, weakly plastic; 1/*H* > 0.75, plastic. The 1/*H* is a useful tool that quantifies the stoichiometric homeostasis of submerged macrophytes^10^.

### Statistical analysis

All elements concentrations were log_10_-transformed before analyses to improve the data normality. The tissue element concentrations were averaged at species or lake-species level. Canonical discriminant analyses (CDAs) were conducted to compare multielement composition among groups (lake trophic level and submerged macrophytes taxa) and identify elements with the highest discriminatory power, or those that most effectively distinguish submerged macrophytes in their groups. Twenty lakes were grouped by hierarchical clustering based on lake trophic level before CDA. We used Pearson correlation coefficient to measure the similarity among 20 lakes. These analyses tested lake trophic or taxonomic differences given multiple element concentrations. The goal of CDA was to find linear combinations of multiple variables that maximize differences among groups^8^. Canonical discriminant analysis plots were done to visually analyze differences in multielement composition among trophic level groups and taxonomic groups. All statistical analyses were conducted using IBM SPSS Statistics V19 (Armonk, USA).

## Author Contributions

W.X. and G.H.L. designed the study and analyzed the data; W.X. and H.P.W. wrote the manuscript; all authors contributed substantially to revisions. W.X., H.P.W., Q.S., B.B.H., H.L. and Z.X.W collected and determined samples.

## Additional Information

**How to cite this article**: Xing, W. *et al.* Multielement stoichiometry of submerged macrophytes across Yunnan plateau lakes (China). *Sci. Rep.* **5**, 10186; doi: 10.1038/srep10186 (2015).

## Supplementary Material

Supplementary Information

## Figures and Tables

**Table 1:** Stoichiometric homeostasis coefficient (1/*H*_X:C_) for submerged macrophytes taxa in Yunnan plateau lakes.

	**1/*H***	**1/*H***	**1/*H***	**1/*H***	**1/*H***	**1/*H***	**1/*H***	**1/*H*_**Mn:C**_**	**1/*H*_**Mo:C**_**	**1/*H***	**1/*H***	**1/*H*_**Si:C**_**	**1/*H*_**Zn:C**_**
Potamogetonaceae	0.956	1.311	−4.516	1.800	14.255	2.656	3.396	1.741	1.171	−0.136	1.627	2.136	1.631
	100*	100	100	93	100	100	100	100	70	100	100	83	100
Hydrocharitaceae	0.797	1.307	−3.465	1.841	−4.640	1.069	1.943	1.749	1.285	−2.088	1.681	1.925	1.661
	25	25	25	24	25	25	25	25	18	25	25	24	25
Haloragaceae	0.912	1.384	−2.807	1.930	7.021	2.081	1.084	1.866	1.173	1.262	1.799	2.499	1.710
	30	30	30	27	30	30	30	30	17	30	30	27	30
Characeae	1.021	1.344	−1.787	1.732	29.290	4.312	1.934	1.586	1.244	1.519	1.678	1.856	1.628
	12	12	12	12	12	12	12	12	6	12	12	12	12
Ceratophyllaceae	0.821	1.064	−1.967	1.656	4.725	0.720	3.215	1.224	1.189	1.926	1.529	2.043	1.474
	10	10	10	10	10	10	10	10	7	10	10	8	10

These data were averaged at taxon level.

^*^number of samples.
