# Supplementary material for: Multielement stoichiometry of submerged macrophytes across Yunnan plateau lakes (China)
Source: Sci Rep. 2015 May 13;5:10186. doi: 10.1038/srep10186 (PMC4429540; doi:10.1038/srep10186)

**Supplementary information**

**Title Page**

**Title: Multielement stoichiometry of submerged macrophytes across Yunnan plateau lakes (China)**

**Authors: Wei Xing, Haoping Wu, Qiao Shi, Beibei Hao, Han Liu, Guihua Liu**

Figure S1 Locations of 20 Yunnan plateau lakes. The locations were mapped using ArcGIS (ArcGIS 9.3.1, ESRI, Redlands, California, USA).

Table S1 Collected submerged macrophyte species in 20 Yunnan plateau lakes.

Table S2 Limnology characteristics of 20 Yunnan plateau lakes^46^. “/” means no data.


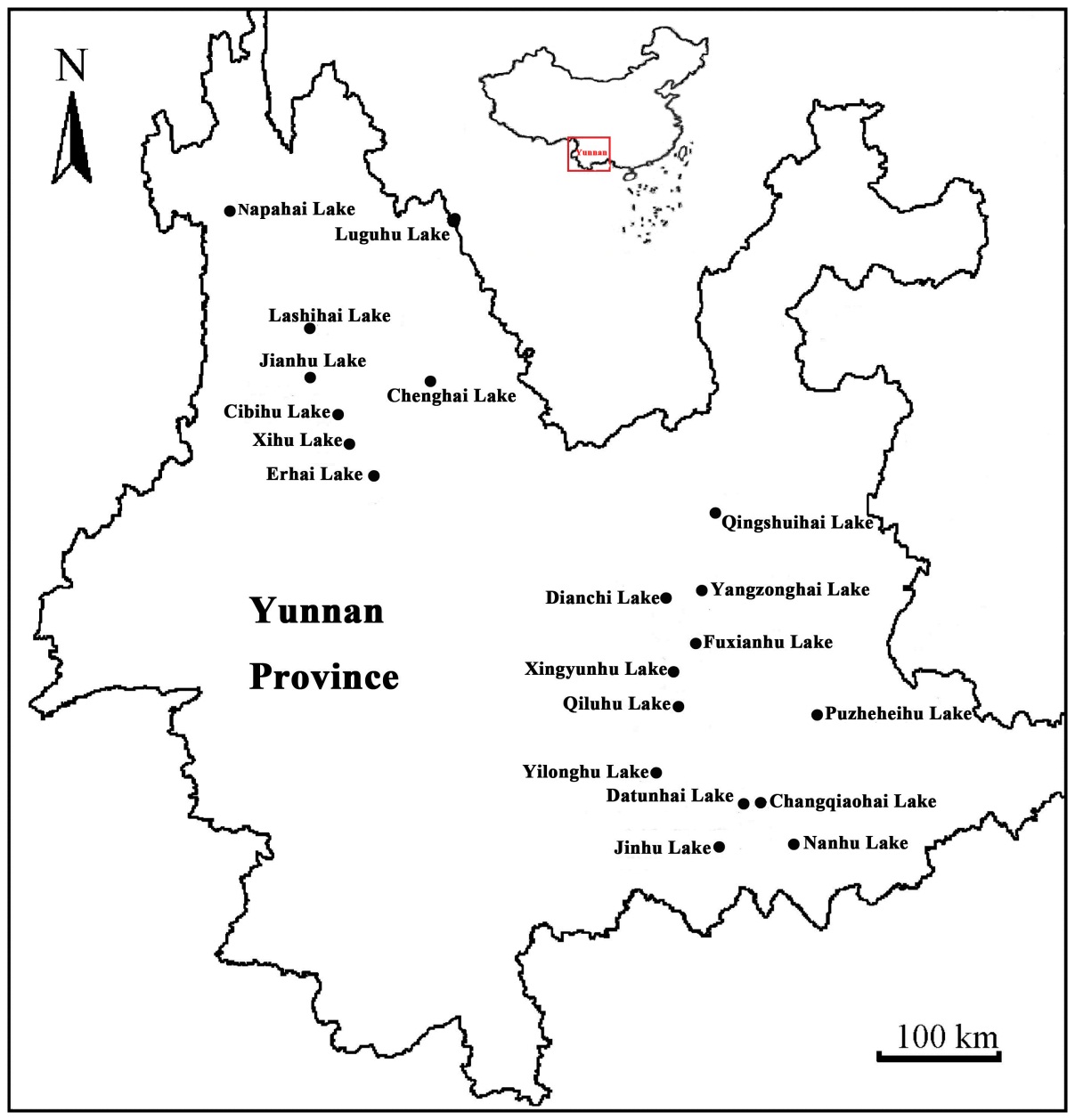


Figure S1

Table S1


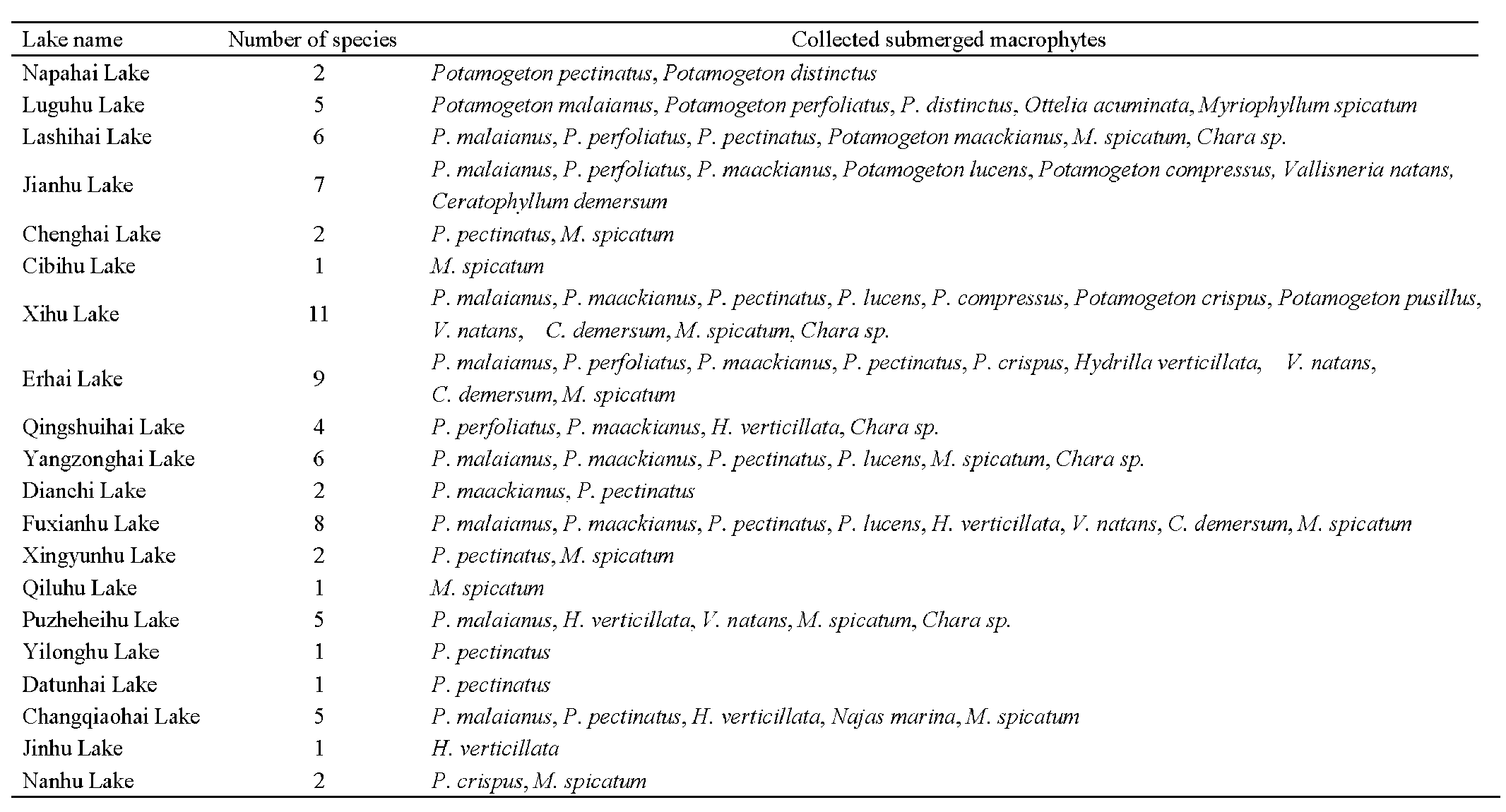


Table S2


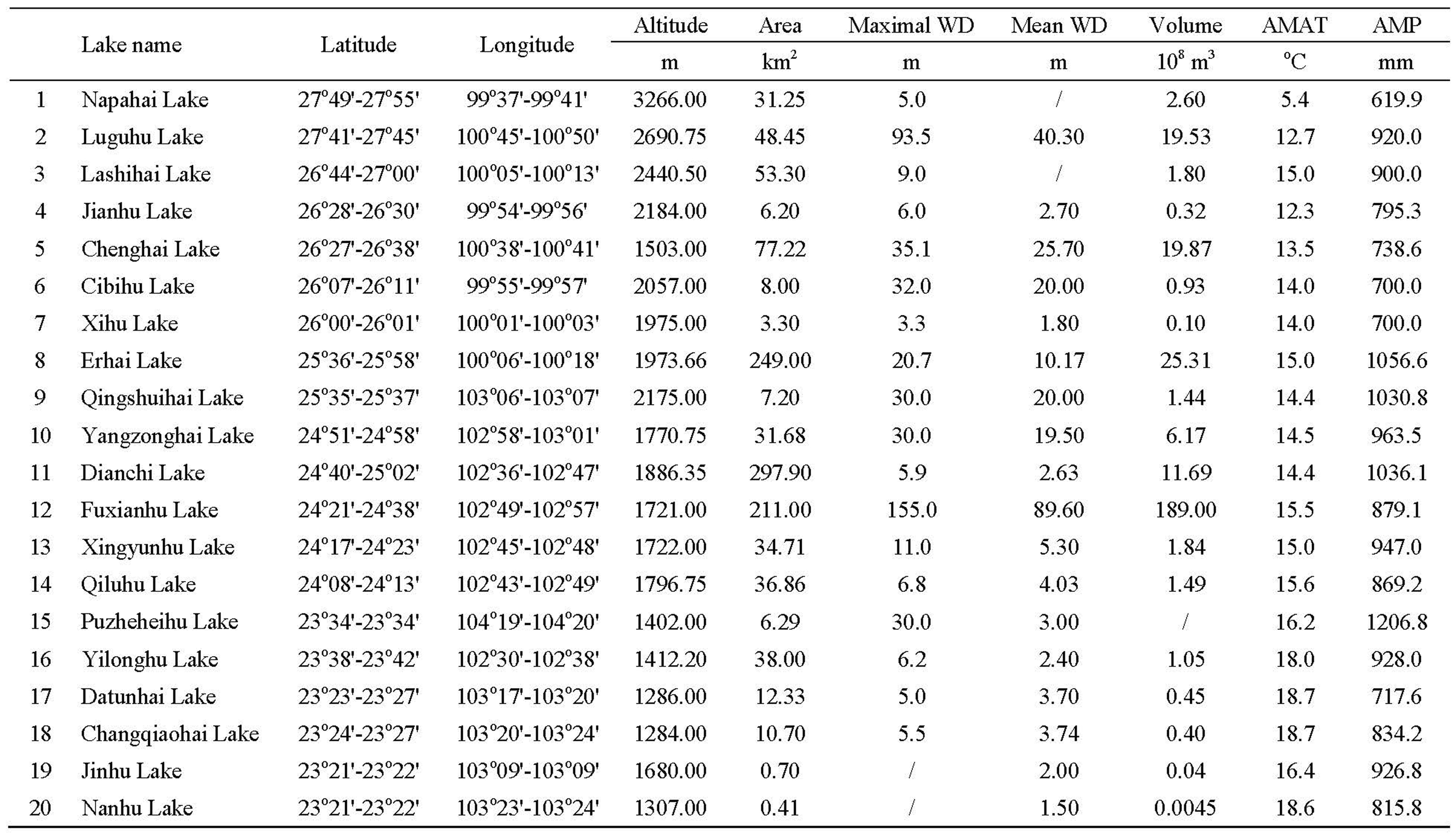

Supplement: Supplementary Information [file srep10186-s1.doc]
